# Daraxonrasib: A Breakthrough in Pancreatic Ductal Adenocarcinoma

**DOI:** 10.7759/cureus.113927

**Published:** 2026-08-04

**Authors:** George S Zacharia, Udesh Pandey, Ornela Thartori, FNU Veena, Samrachana Acharya, Lav Shah, Reyansh Patel, Harish Patel

**Affiliations:** 1 Internal Medicine, BronxCare Health System, New York, USA; 2 Medical Observation, BronxCare Health System, New York, USA; 3 Internal Medicine, Bronx Lebanon Hospital Center, New York, USA

**Keywords:** daraxonrasib, ductal adenocarcinoma, kras, pancreatic cancer, ras inhibitors

## Abstract

Pancreatic cancer is one of the leading causes of cancer-related mortality. Diagnosis at an advanced stage, aggressive tumor biology, and chemoresistance all contribute to the overall dismal prognosis. Somatic, non-hereditary activating mutations involving the proto-oncogene Kirsten rat sarcoma viral oncogene (KRAS) are encountered in the vast majority of patients. KRAS mutations are involved in tumor initiation as well as the progression of pancreatic cancer and are key determinants of prognosis. Beyond pancreatic ductal adenocarcinoma (PDAC), KRAS mutations have been reported in multiple human solid-organ neoplasms. Over the past few decades, targeted molecular therapy has evolved into an indispensable part of oncology. Molecules targeting rat sarcoma viral oncogene (RAS) mutations have gained considerable interest in molecular oncology owing to their pivotal role in common cancers such as non-small cell lung, colon, and pancreatic cancer. One of the recent breakthroughs in PDAC treatment is the discovery of the multi-selective RAS inhibitor specific to its ON conformation: the first of its class, the RAS(ON) inhibitor daraxonrasib. Preclinical and early clinical data confirmed its utility in PDAC, leading to United States Food and Drug Administration permission to expand access treatment protocols for patients with previously treated metastatic PDAC in May 2026. This narrative review summarizes the published literature on the safety and efficacy of daraxonrasib in PDAC, based on a comprehensive PubMed literature search. As of June 2026, the utility of daraxonrasib is limited to metastatic PDAC as a second-line agent in patients who failed or were intolerant of first-line chemotherapy. An ongoing trial is evaluating the efficacy of daraxonrasib as a first-line targeted therapy in metastatic PDAC. Further, large-scale, blinded, multicenter randomized trials are required to confirm the efficacy and safety of daraxonrasib in PDAC and other solid-organ neoplasms. The orally administered daraxonrasib could be a potential game changer in the treatment of PDAC, which is otherwise considered one of the least chemotherapy-amenable human cancers.

## Introduction and background

Pancreatic ductal adenocarcinoma (PDAC) or pancreatic cancer is one of the leading causes of cancer-related mortality across the globe. According to the American Cancer Society (ACS), PDAC accounts for 3% of all cancers and 8% of all cancer-related mortalities in the United States [1]. It is estimated that about 67,530 people will be diagnosed with PDAC and 52,740 will die from it in 2026 [1]. More frightening is the prognosis of PDAC; with an estimated five-year survival rate across all stages, it is only 13% [2]. The factors contributing to poor survival include an advanced stage at diagnosis, aggressive tumor biology, and treatment resistance, as well as rapid clinical decline. Kirsten rat sarcoma viral oncogene (KRAS) mutations are identified in more than 90% of patients with PDAC and are believed to contribute to its aggressive nature and resistance [3]. This has led to increasing interest in KRAS inhibition for the management of PDAC. Multiple KRAS inhibitors have been evaluated in the treatment of solid organ cancers, including PDAC. However, molecules that target the OFF conformation of KRAS or are mutation-selective inhibitors often encounter rapid evolution of drug resistance through different mechanisms. One of the frequent mechanisms of RAS inhibitor failure or resistance is the presence of multiple KRAS mutations, either simultaneous or acquired over time [4]. This has left an unmet need for novel KRAS inhibitors with better efficacy and less risk of resistance.

Daraxonrasib (drug code: RMC-6236), a multiselective rat sarcoma viral oncogene (RAS) inhibitor, has broad-spectrum activity against wild-type and various mutated KRAS oncogenes [4]. Recent trials demonstrated favorable outcomes in patients with PDAC treated with daraxonrasib, leading to United States Food and Drug Administration (USFDA) permission for an expanded-access treatment protocol for patients with previously treated metastatic PDAC in May 2026 [5]. Daraxonrasib is largely regarded as a breakthrough in the management of advanced PDAC, though the efficacy needs to be confirmed with further studies. This narrative review aims to provide a comprehensive overview of the published literature on daraxonrasib in PDAC. The review examines the pharmacological properties and mechanism of action of daraxonrasib and summarizes the available evidence on its efficacy and safety from preclinical and clinical studies published to date.

## Review

Methods

A systematic literature search was performed using the PubMed and MEDLINE databases from inception through July 2026. The following Boolean search string was employed: ("daraxonrasib" OR "RMC-6236") AND ("pancreatic ductal adenocarcinoma" OR "pancreatic neoplasms" OR "PDAC" OR "pancreatic cancer"). No language or date restrictions were applied. Inclusion criteria included original research articles, clinical trial reports, conference abstracts, translational studies, and review articles addressing the use of daraxonrasib (RMC-6236) in PDAC or pancreatic cancer; studies reporting on preclinical, pharmacological, or clinical outcomes of daraxonrasib in RAS-mutant pancreatic cancer; and peer-reviewed publications and indexed conference abstracts. Duplicate publications reporting identical datasets; editorials, commentaries, letters to the editor, and opinion pieces without original data; studies evaluating daraxonrasib exclusively in non-pancreatic malignancies without relevant pancreatic cancer data; and articles not available in full text were excluded. The search results were independently screened by two reviewers. Studies were categorized by type (preclinical/translational, phase I/II clinical trials, phase III clinical trials, conference abstracts, and review articles) and summarized accordingly. A systematic analysis was not feasible because the published literature was very limited and heterogeneous. As the drug is currently in advanced trials and clinical development, a narrative design should provide an overview of the drug. The narrative design is subject to inherent selection bias, a major limitation of the current review.

KRAS mutation and pancreatic cancer

**Figure 1:**
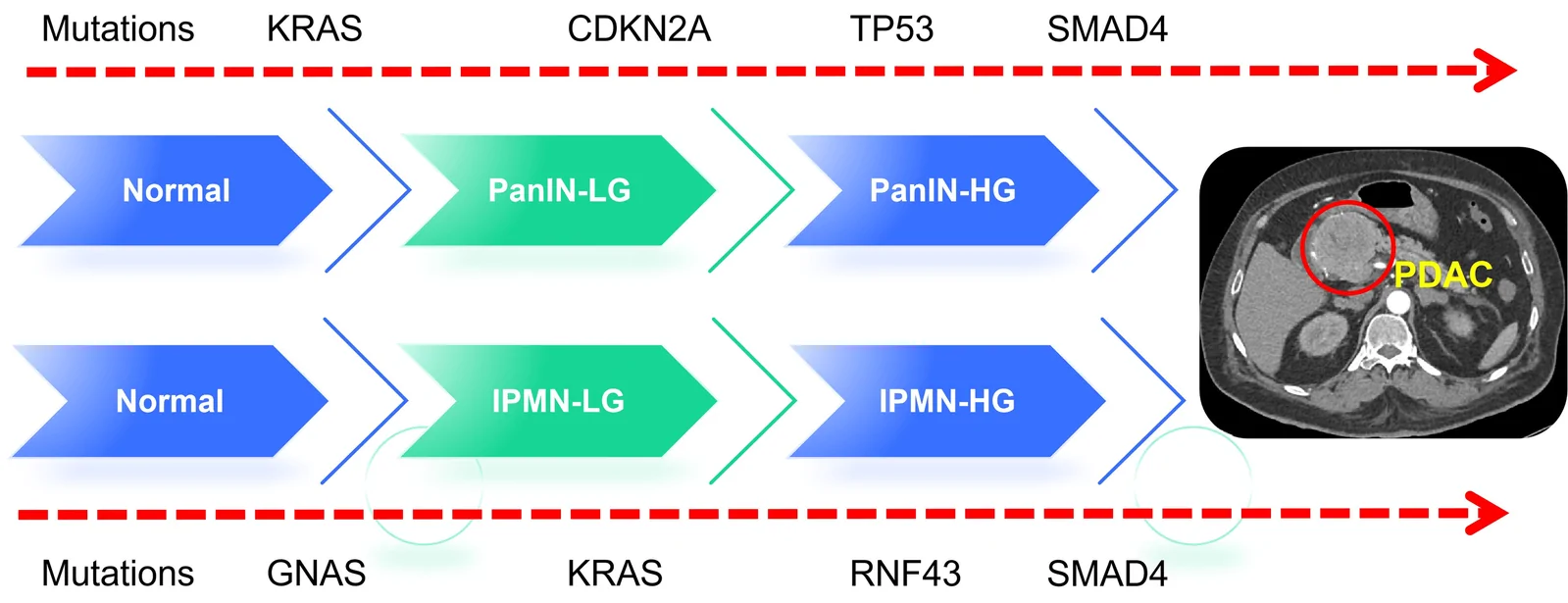
Molecular pathways and progression of precursor lesions to pancreatic ductal adenocarcinoma IPMN: Intraductal papillary mucinous neoplasm; PanIN: Pancreatic intraepithelial neoplasia; LG: Low-grade; HG: High-grade; PDAC: Pancreatic ductal adenocarcinoma; KRAS: Kirsten rat sarcoma viral oncogene; CDKN2A: Cyclin-dependent kinase inhibitor 2A; TP53: Tumor protein 53; SMAD4: Mothers against decapentaplegic homolog 4; GNAS: Guanine nucleotide-binding protein, alpha stimulating activity; RNF43: Ring finger protein 43. Figure created using Microsoft PowerPoint

KRAS mutations are the most frequently reported genetic alterations in patients with PDAC, occurring in more than 90% of cases. The KRAS mutations associated with PDAC are typically somatic-acquired or sporadic, not inherited. PDAC arises from either of the two precursor lesions: pancreatic intraepithelial neoplasia (PanIN) or intraductal papillary mucinous neoplasm (IPMN) [6]. PanINs are microscopic lesions, typically arising from the small ductal or acinar epithelial cells, while IPMNs are macroscopic lesions arising from the larger ducts. PanINs are more frequently the precursors of PDAC than IPMNs; studies report that more than 80% of PDACs arise from PanINs [7]. The Baltimore Consensus (2014) classified both PanIN and IPMN into a two-tier system: low-grade and high-grade. The intermediate grades were merged into the low grades. High grade is sometimes referred to parenthetically as carcinoma in situ [8]. KRAS mutations are identified as the earliest mutation, believed to be the “driver mutation” in the PanIN pathway, with >95% of PanINs harboring KRAS mutations [9]. The other mutations involved in the progression from PanIN to PDAC include cyclin-dependent kinase inhibitor 2A (CDKN2A), tumor protein 53 (TP53), and mothers against decapentaplegic homolog-4 (SMAD4) (Figure 1). Guanine nucleotide-binding protein, alpha stimulating activity (GNAS) mutations are typically the earliest genetic changes in IPMN, while KRAS mutations also contribute to its progression; they are reported in up to 65% of patients [9].

Multiple KRAS-activating mutations have been reported to date; most involve codon 12 of exon 2, resulting in a conformational change at the guanosine triphosphate (GTP)-binding site (Table 1) [10,11]. This leads to impaired guanosine triphosphatase (GTPase) activity, which is responsible for converting active GTP to inactive guanosine diphosphate (GDP). Activated RAS activates multiple downstream pathways and transcription factors, leading to PDAC initiation, proliferation, angiogenesis, immune evasion, and metastasis [9-12]. The phosphoinositide 3-kinase/protein kinase B (PI3K/AKT) and rapidly accelerated fibrosarcoma/mitogen-activated protein kinase/extracellular signal-regulated kinase (RAF/MAPK/ERK) signaling pathways play critical roles in PDAC initiation and progression. KRAS mutations also contribute to the metabolic reprogramming of glucose, glutamine, fatty acids, acetyl-CoA, branched-chain amino acids, and reactive oxygen species, thereby promoting tumor development and progression [12]. KRAS mutation modulates immune pathways: it upregulates regulatory T cells, tumor-associated macrophages, and the helper T cell (Th2/Th1) ratio, while downregulating Th1 and cluster of differentiation (CD)8+ T cells, thereby allowing tumor cells to survive [12]. Furthermore, KRAS mutations are often believed to be non-treatable with drugs, highly chemoresistant, and associated with the upregulation of the antioxidant factor nuclear factor erythroid 2-related factor 2 (NRF2) and tumor heterogeneity. KRAS mutant variants have been linked to distant metastasis, poor survival, and a poor prognosis [12].

**Table 1:** KRAS mutations in pancreatic ductal adenocarcinoma KRAS: Kirsten rat sarcoma viral oncogene

KRAS mutations [10,11]	
G12D (Glycine to Aspartate)	35% - 42%
G12V (Glycine to Valine)	30% - 32%
G12R (Glycine to Arginine)	14% - 17%
G12C (Glycine to Cysteine)	1% - 2%
Q61 mutations	5-6%

Daraxonrasib

Mechanism of Action

**Figure 2:**
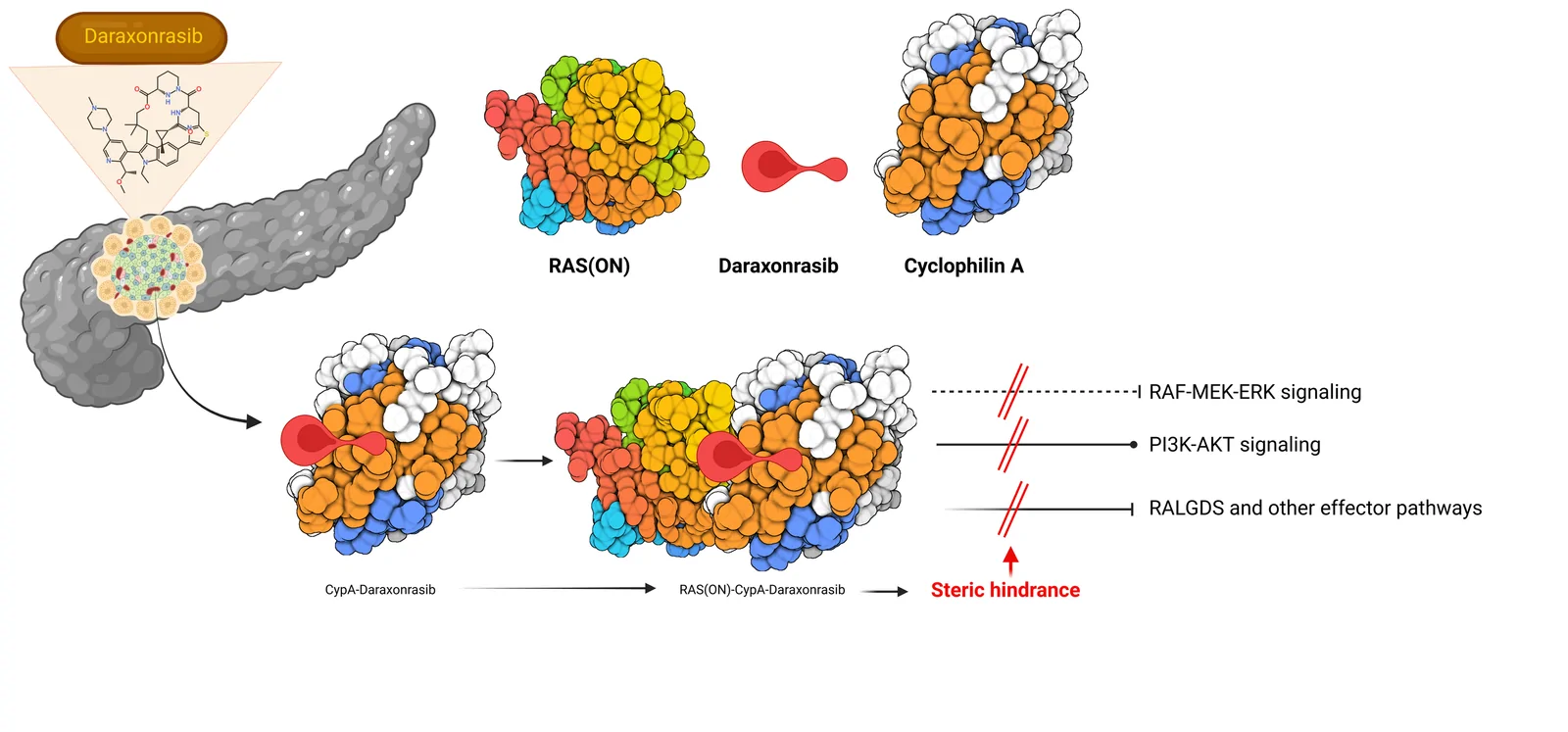
Mechanism of action of daraxonrasib Daraxonrasib binds to CypA, and the resulting CypA–daraxonrasib complex engages activated RAS(ON), sterically hindering interactions between RAS and its downstream effector proteins. This disruption suppresses multiple oncogenic signaling cascades, including the RAF–MEK–ERK (MAPK) pathway, the PI3K–AKT pathway, and RALGDS-mediated signaling, thereby inhibiting tumor cell proliferation and survival. By targeting the active state of RAS rather than a specific mutant isoform, daraxonrasib has the potential to inhibit signaling driven by a broad spectrum of RAS alterations. CypA: Cyclophilin A; MAPK: Mitogen-activated protein kinase; PI3K: Phosphoinositide 3-kinase; AKT: Protein kinase B; RAF: Rapidly accelerated fibrosarcoma kinase; MEK: Mitogen-activated protein kinase; ERK: Extracellular signal-regulated kinase; RALGDS: Ral guanine nucleotide dissociation stimulator Figure created in BioRender. Zacharia, G. (2026) https://BioRender.com/ q0jwujm).

Daraxonrasib is an inhibitor of the “ON” (GTP-bound) conformations of KRAS, Harvey RAS (HRAS), and neuroblastoma RAS (NRAS), both mutant and wild-type strains: RAS(ON) inhibitor. The molecule within the cell interacts with the intracellular chaperone protein cyclophilin-A to form a binary complex. The binary complex, in turn, interacts with RAS(ON), forming a large tricomplex [4]. This complex sterically blocks the RAS interaction with downstream molecules and disrupts downstream activation of KRAS pathways, thereby negating or attenuating the effects of activated KRAS (Figure 2) [4]. Preclinical models have demonstrated intratumoral accumulation of the drug with sustained RAS/MAPK suppression [13].

The conventional KRAS inhibitors target the ‘OFF’ conformation of KRAS (GDP-bound RAS) and/or specific KRAS mutations. These molecules are often effective initially, but they develop resistance through multiple mechanisms. Mechanisms include the development of additional KRAS mutations, KRAS amplification, drug-binding site alteration, activation of upstream or downstream regulators, etc. Acquiring additional mutations will diminish the efficacy of mutation-specific inhibitors, such as sotorasib or adagrasib, which are specific inhibitors of G12C-mutated KRAS. Additionally, RAS-OFF inhibitors are largely ineffective against the ON targets, which drive carcinogenesis. An alteration in the inhibitor binding site leads to drug resistance due to the failure of interaction between KRAS and the inhibitor [4]. Daraxonrasib overcomes the limitations of traditional RAS inhibitors with its broad-spectrum activity, being effective against KRAS, HRAS, NRAS, wild-type, and multiple mutant variants of RAS. Because there is no covalent bonding between daraxonrasib and RAS, binding-site alterations are unlikely to confer drug resistance as well.

Pharmacokinetic Profile

The drug is bioavailable after oral administration. Preclinical models report oral bioavailability ranging from 24% to 33%, varying across species [4]. Intestinal absorption of the drug is mediated by adenosine triphosphate (ATP)-binding cassette transporters and organic anion transporters (OATPs) [14,15]. In animal models, the time to maximum concentration (Tmax) varied from 0.7 to 4.0 hours [4]. The phase I/II trial by Wolpin et al. reported a Tmax of 1.6 to 3.9 hours [14]. The trial also reported a dose-dependent increase in steady-state whole-blood drug concentration at the evaluated oral doses, ranging from 10 mg/day to 400 mg/day [14]. Hepatic uptake of the drug is mediated by OATP, and its clearance is mediated by the cytochrome P450 (CYP) system, specifically CYP3A4 [15]. The half-life ranged from 2.4 hours to 5.3 hours in different animal models [4]. Wolpin et al. estimated a terminal half-life of 11.8 hours [14].

Preclinical Evidence

The seminal preclinical trial of RMC-6236 (later named daraxonrasib) by Jiang et al. reported efficacy in RAS-driven cancer in vitro and in vivo models. A broad spectrum of antitumor activity against KRAS-G12X-mutated PDAC, colorectal cancer, and non-small cell lung cancer was observed at a daily dose of 25 mg/kg for up to 90 days. A statistically significant reduction in tumor volume was observed with RMC-6236 compared with the control group [11].

Trials combining RAS(ON) inhibitors with other targeted molecular agents are also being evaluated for improved outcomes. The preclinical trial by Liaki et al., which combined daraxonrasib, afatinib (Epidermal Growth Factor Receptor/Human Epidermal Growth Factor Receptor 2 (EGFR/HER2) kinase inhibitor), and SD36 (STAT3 Degrader 36 (STAT: Signal Transducer and Activator of Transcription)), reported superior outcomes [16]. Yet another study evaluated the selective proliferating cell nuclear antigen (PCNA) inhibitor AOH1996, in combination with KRAS inhibitors, and demonstrated synergistic effects in mutant models [17]. Orlen et al. demonstrated the synergistic benefits of combining doraxonrasib with anti-programmed cell death protein 1 (anti-PD1) therapy. Mouse models treated with a combination of daraxonrasib and anti-PD1 had more durable responses than either agent alone [18]. 

Clinical Evidence

The Phase I/II trial by Wolpin et al. included 168 patients with advanced PDAC who had failed or were intolerant to conventional chemotherapy; 89% had RAS-G12 mutations. Patients were administered daraxonrasib orally at doses of 10 mg to 400 mg per day. A dose of 300 mg/day was found to be superior to lower doses. At this dose, for RAS-G12 mutations, the objective response rate was 35% (95% CI: 17 to 56), the median time to response was 2.6 months, and the duration of response was 8.2 months. After 17 months of follow-up, the median progression-free survival and overall survival were 8.5 months and 13.1 months, respectively, for the subgroup of patients harboring RAS-G12 mutations. The Kaplan-Meier estimated overall survival rates were 100% at six months and 89% at nine months. Patients with any RAS mutation had a poorer response than those with RAS-G12 mutations [14]. The study, though, resulted in meaningful objective responses and promising survival outcomes; the findings should be interpreted within the context of an early-phase, single-arm trial. The absence of a randomized comparator, a relatively small sample size, and limited follow-up restrict definitive conclusions regarding comparative efficacy and long-term clinical benefit. A further limitation is the heterogeneous nature of the study population. Patients differed with respect to KRAS mutation subtype, prior chemotherapy regimens, and the number of treatment lines received before initiating daraxonrasib.

**Figure 3:**
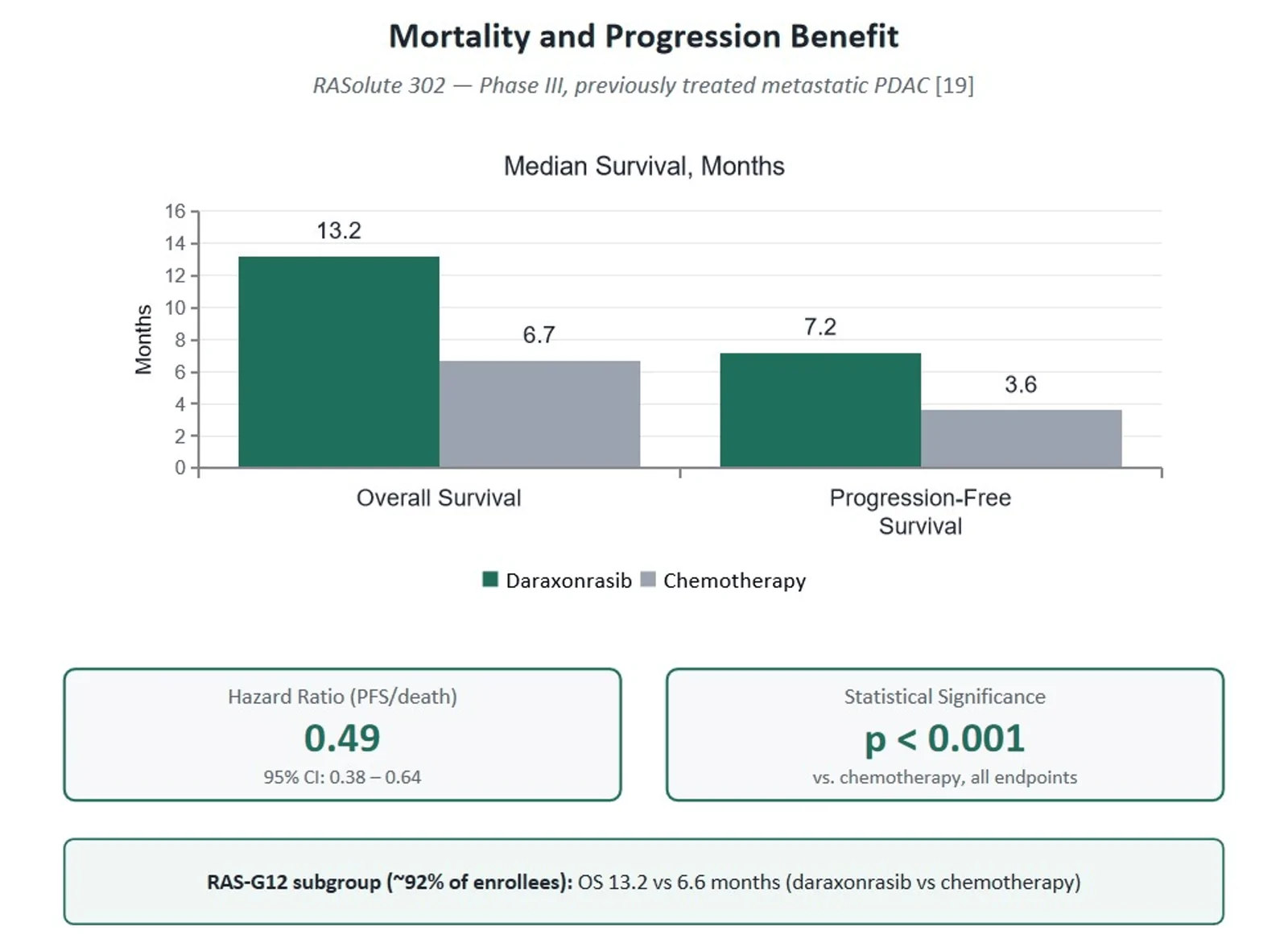
Daraxonrasib in metastatic pancreatic ductal adenocarcinoma PDAC: Pancreatic ductal adenocarcinoma; PFS: Progression free survival; CI: Confidence interval; OS: Overall survival

The RASolute 302 Phase III randomized, open-label, multicenter trial enrolled previously treated patients with metastatic PDAC to receive oral daraxonrasib, 300 mg daily, or other chemotherapeutic agents. Among the study population, nearly 92% harbored RAS-G12 mutations. The median survival in the daraxonrasib group was 13.2 months overall and 13.2 months in those with RAS-G12 mutations, while with chemotherapy it was 6.7 months overall and 6.6 months in those with RAS-G12 mutations. Progression-free survival was also doubled in patients receiving daraxonrasib compared with chemotherapy (7.2 months versus 3.6 months). The hazard ratio for disease progression or death was 0.49 (95% confidence interval (CI): 0.38 to 0.64). All favorable responses were statistically significant compared with chemotherapy (Figure 3) [19]. The trial is limited by its open-label design and the use of investigator-selected chemotherapy as the comparator, which could have introduced treatment variability.

The ongoing RASolute 303 Phase III randomized, multicenter trial enrolls treatment-naive patients with metastatic PDAC to receive daraxonrasib monotherapy, daraxonrasib plus chemotherapy, or chemotherapy alone [20]. Early data reveal objective response rates of 47% in patients receiving daraxonrasib monotherapy and 58% in those receiving combination therapy [21]. The final results of the study are still awaited. The RAS 304 Phase III randomized, multicenter trial is currently enrolling patients with resected PDAC who have completed adjuvant or neoadjuvant therapy to receive daraxonrasib versus observation only [22].

Adverse Effects

The most commonly reported adverse effects with daraxonrasib include rash, diarrhea, stomatitis, nausea, vomiting, and fatigue [19]. Table 2 compares the reported side effects of daraxonrasib across the phase I/II and phase III trials. Treatment-related side effects were reported in almost all patients with daraxonrasib or chemotherapy in the Phase III trial; however, grade ≥3 adverse events were more frequent with chemotherapy (Table 3). Daraxonrasib was associated with predominantly dermatologic and gastrointestinal adverse events, particularly rash (85.5%), stomatitis (53.1%), diarrhea (58.1%), nausea (46.5%), and vomiting (36.9%), while chemotherapy caused substantially more hematologic toxicity. Dose interruptions occurred at similar rates, but dose reductions were substantially less common with daraxonrasib (36.1% vs. 57.5%). Rash and stomatitis were the primary causes of dose reduction with daraxonrasib [19].

**Table 2:** Adverse effect profile of daraxonrasib in clinical trials AE: Adverse effect(s); NR: Not reported; AST: Aspartate aminotransferase; ALT: Alanine aminotransferase

Adverse Event	Wolpin et al. [14]	RASolute 302 [19]
Any treatment-related AE	96%	97.9%
Grade ≥3 treatment-related AE	30%	43.6%
Serious treatment-related AE	6%	10.8%
Treatment discontinuation due to AE	1%	1.2%
Dose interruption	37%	56.0%
Dose reduction	21%	36.1%
Rash	88% (Grade ≥3: 7%)	85.5% (Grade ≥3: 13.7%)
Diarrhea	46% (3%)	58.1% (5.4%)
Stomatitis/Mucositis	40% (4%)	53.1% (12.0%)
Nausea	42% (0%)	46.5% (2.1%)
Vomiting	31% (0%)	36.9% (0.4%)
Fatigue	20% (2%)	23.2% (3.7%)
Decreased appetite	11% (1%)	17.0% (1.2%)
Paronychia	15% (0%)	16.6% (0%)
Dry skin	10% (0%)	13.3% (0%)
AST increased	NR	12.4% (2.5%)
ALT increased	NR	9.5% (1.7%)
Anemia	NR	18.3% (4.1%)
Thrombocytopenia	NR	10.0% (2.1%)
Neutropenia	NR	7.5% (1.7%)
Asthenia	NR	10.8% (0.4%)
Peripheral edema	NR	8.7%

**Table 3:** Comparison of adverse effects with daraxonrasib and chemotherapy AE: Adverse effect(s). Reference: RASolute 302 trial [19]

Safety Profile	Daraxonrasib	Chemotherapy
Any adverse event	100%	97.7%
Grade ≥3 adverse event	61.8%	69.6%
Any treatment-related AE	97.9%	93.5%
Grade ≥3 treatment-related AE	43.6%	57.5%
Serious treatment-related AE	10.8%	18.7%
Grade 5 treatment-related AE	0.4%	0%
Dose modification	56.8%	71.5%
Dose interruption	56.0%	55.1%
Dose reduction	36.1%	57.5%
Treatment discontinuation	1.2%	11.2%

In summary, daraxonrasib has evoked considerable enthusiasm in oncology, with preclinical and early clinical trials reporting favorable outcomes in PDAC, one of the most challenging, aggressive, and poorly responsive human malignancies. The current evidence is limited to patients with metastatic PDAC, in whom first-line agents failed or were intolerable. It remains unclear whether the observed clinical benefits will translate into comparable efficacy when daraxonrasib is used as first-line therapy for advanced or metastatic PDAC. The severe adverse effects reported in nearly half of the subjects are another major caveat. The ongoing RASolute 303 trial is expected to shed light on the utility of daraxonrasib as a first-line agent in metastatic PDAC. Further trials are required to evaluate its utility in non-metastatic PDAC. Also, drug tolerance, long-term safety, and the durability of response are largely unknown.

## Conclusions

Daraxonrasib is a multi-selective RAS inhibitor, selective for the activated RAS conformation: RAS(ON) inhibitor. By targeting RAS-driven signaling, one of the fundamental drivers of PDAC, daraxonrasib offers a new therapeutic horizon for a malignancy that has historically been difficult to treat and has a dismal prognosis. The encouraging results from preclinical studies and recent early-phase clinical trials, particularly the improvements in survival observed in patients with metastatic PDAC treated with daraxonrasib as a second-line therapy, have garnered considerable interest within the oncology community. However, at this time, the clinical experience with daraxonrasib remains relatively early. Questions regarding long-term safety, the durability of the response, resistance mechanisms, and its efficacy in earlier stages of disease have yet to be fully answered. Ongoing and future studies will be critical in defining its place in the treatment algorithm and determining whether its benefits can be extended through combination approaches or earlier intervention.
